# Genetic Rodent Models of Obesity-Associated Ovarian Dysfunction and Subfertility: Insights into Polycystic Ovary Syndrome

**DOI:** 10.3389/fendo.2016.00053

**Published:** 2016-06-07

**Authors:** Isabel Huang-Doran, Stephen Franks

**Affiliations:** ^1^Institute of Reproductive and Developmental Biology, Department of Surgery and Cancer, Imperial College London, Hammersmith Hospital, London, UK

**Keywords:** androgen, fertility, insulin resistance, mouse models, obesity, PCOS

## Abstract

Polycystic ovary syndrome (PCOS) is the most common endocrinopathy affecting women and a leading cause of female infertility worldwide. Defined clinically by the presence of hyperandrogenemia and oligomenorrhoea, PCOS represents a state of hormonal dysregulation, disrupted ovarian follicle dynamics, and subsequent oligo- or anovulation. The syndrome’s prevalence is attributed, at least partly, to a well-established association with obesity and insulin resistance (IR). Indeed, the presence of severe PCOS in human genetic obesity and IR syndromes supports a causal role for IR in the pathogenesis of PCOS. However, the molecular mechanisms underlying this causality, as well as the important role of hyperandrogenemia, remain poorly elucidated. As such, treatment of PCOS is necessarily empirical, focusing on symptom alleviation. The generation of knockout and transgenic rodent models of obesity and IR offers a promising platform in which to address mechanistic questions about reproductive dysfunction in the context of metabolic disease. Similarly, the impact of primary perturbations in rodent gonadotrophin or androgen signaling has been interrogated. However, the insights gained from such models have been limited by the relatively poor fidelity of rodent models to human PCOS. In this mini review, we evaluate the ovarian phenotypes associated with rodent models of obesity and IR, including the extent of endocrine disturbance, ovarian dysmorphology, and subfertility. We compare them to both human PCOS and other animal models of the syndrome (genetic and hormonal), explore reasons for their discordance, and consider the new opportunities that are emerging to better understand and treat this important condition.

## Introduction

The association between obesity, insulin resistance (IR), type II diabetes (T2DM), cardiovascular disease, and non-alcoholic fatty liver disease is well-established in the literature, discussed commonly in the clinic, and subject to intensive investigation in laboratories worldwide (1, 2). Perhaps less recognized is obesity’s association with ovarian dysfunction, most commonly in the form of polycystic ovary syndrome (PCOS). Diagnostic criteria of PCOS incorporate three key features: biochemical and/or clinical evidence of androgen excess (including acne, hirsutism, and alopecia), ovarian dysfunction or anovulation (manifesting as absent or irregular menstruation), and the appearance of multiple peripheral cysts on ovarian ultrasonography (3). Rarer causes of raised androgen levels (such as an androgen-producing tumor) are first excluded. Metabolic dysfunction is common but not invariable in women with PCOS and so, although cross-sectional and longitudinal studies support a significant role for IR in the etiology of PCOS, diagnostic criteria do not currently incorporate metabolic parameters. Nevertheless, not only is PCOS the most common cause of anovulatory infertility and menstrual irregularity but (since it often manifests in the second and third decades) young women with PCOS also represent a large, identifiable group who may be at increased risk of metabolic (4–6) and cardiovascular diseases (7–10). Indeed, PCOS is a strong predictor of future T2DM (11). Women with PCOS therefore represent an important target for research and prevention.

The heterogeneous nature of PCOS, along with a lack of consensus over precise diagnostic criteria, has complicated efforts to understand its pathogenesis. Familial clustering studies and monozygotic twin concordance reveal an important genetic predisposition to the syndrome. Genetic variants identified from candidate gene screening and genome-wide association studies implicate insulin, growth factor, and gonadotrophin signaling, cellular proliferation, and DNA repair pathways; however, they so far account for less than 10 percent of the syndrome’s heritability (12). The presence of PCOS-like features in animals exposed prenatally to androgens suggests that PCOS may have important developmental origins (13). Genetic and developmental influences likely interact with environmental factors in adolescence and adulthood to produce the complex physiological dysregulation that characterizes this syndrome.

**Figure 1:**
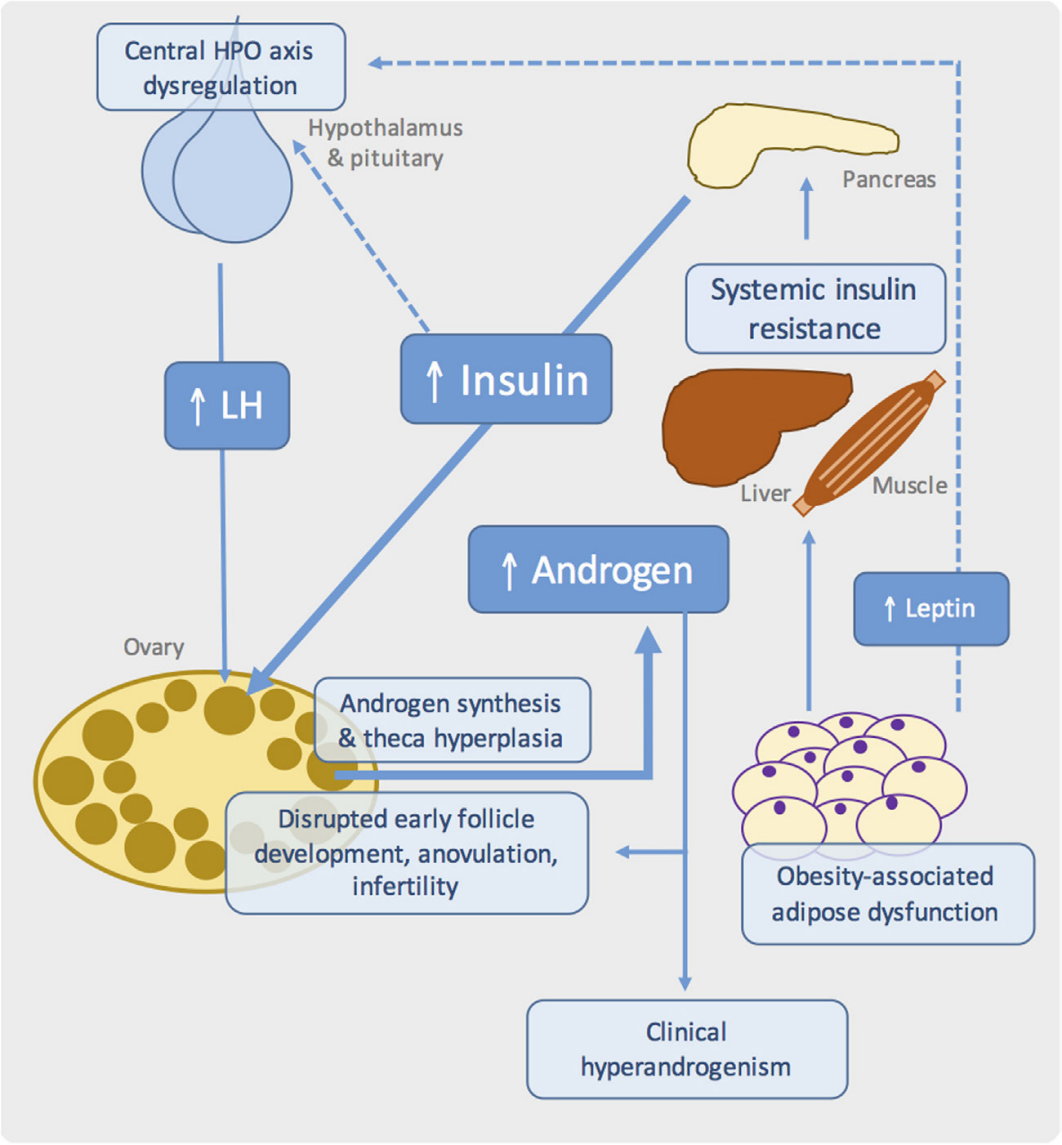
**Proposed pathogenic mechanisms in obesity-associated ovarian dysfunction and subfertility**. Schematic showing the major metabolic and reproductive pathways involved in PCOS. Systemic insulin resistance, commonly due to adipose tissue dysfunction in the context of obesity, results in compensatory hyperinsulinemia. At the ovary, insulin synergizes with luteinizing hormone (LH) to drive androgen synthesis. Disrupted insulin, growth factor, gonadotrophin, and sex steroid signaling in the ovary leads to failure of follicle development and ovulation. Genetic and developmental influences are also likely to play an important role.

Hormonal models, in which rodents, sheep, and non-human primates are treated during development or postnatally with androgens (testosterone, DHT, or DHEA), estrogens, aromatase inhibitors, or antiprogestins, are widely employed in PCOS research (14–19). Genetic rodent models offer a complementary albeit underutilized strategy in this field, allowing the contribution of individual genes to be evaluated on “clean” genetic backgrounds and providing tractable and affordable models in which to interrogate disease pathways (14, 20–23). Their value, however, depends on the fidelity of the model to human physiology and disease and the relevance of single-gene perturbations. After summarizing some main concepts relating to the pathogenesis of PCOS (Figure 1), we describe key rodent models relevant to the study of ovarian dysfunction in metabolic diseases (Table 1) and explore why their interpretation may be more complicated than initially apparent.

**Table 1:** **Reproductive features of rodent models of obesity and insulin resistance**.

**Model**	**Body weight**	**Associated metabolic phenotype**	**Sex steroids**	**Gonadotrophins**	**Fertility**	**Ovarian morphology**	**Menstrual cyclicity**	**Comments**	**Key reference**
Human PCOS	↑	IR, ↑ insulin, T2DM, ↑ lipids (independent of BMI)	↑ T	↑ LH	Subfertile	Multiple small, cortical cysts due to follicular arrest, follicular atresia, ↓ CLs	Oligo-/amenorrhea	Insulin sensitizers improve menstrual regularity and hyperandrogenism.	(2)
				↓ FSH					
High-fat diet mouse	↑	IR, ↑ insulin, ↑ FBG	N/R	↑ LH	Subfertile	Diminished follicular development, old CLs	Irregular	Fertility restored after exogenous gonadotrophin (suggests HH).	(24, 25)
				↑ FSH					
*ob*/*ob* mouse	↑	IR, ↑ insulin, ↑ FBG, glucose intolerance, ↑ lipids	↑ T	LH →	Infertile	Ovarian atrophy, follicular atresia, ↓ CLs, no cysts	Acyclic, anovulatory	Ovarian interstitial cytolipema. Phenotype rescued with leptin.	(26–28)
			↑ E2	↓ FSH					
*db*/*db* mouse	↑	IR, ↑ insulin, ↑ FBG, glucose intolerance	↓ E2/P	N/R	Subfertile	Ovarian atrophy, progressive follicular atresia	Irregular	Ovarian interstitial cytolipema.	(29–32)
Zucker rat	↑	IR, ↑ insulin, ↑ FBG, glucose intolerance	↓ T	LH →	Subfertile	↑ total follicle numbers, follicular atresia	Irregular (prolonged diestrus)		(33, 34)
			↓ E2	FSH →					
Koletsky (JCR:LA-cp) rat	↑	↑ insulin, ↑ FBG, ↑ lipids	↑ T	N/R	Subfertile	Ovarian atrophy, cystic follicles, follicular atresia, thin GC layer, ↓ CLs	Irregular		(35, 36)
			E2 →						
NZO rat (polygenic)	↑	IR, ↑ insulin, ↑ FBG, ↑ lipids	T →	↓ LH	Subfertile	↑ ovarian volume, ↑ total follicle numbers, follicular atresia, ↓ CLs, no cysts	Irregular		(37–39)
			↓ E2	FSH →					
Neuron-specific IR deletion (mouse)	↑	Mild IR, ↑ insulin, ↑ TGs	N/R	N/R	Subfertile	Large, luteinized ovarian cysts, thecal-interstitial hyperplasia, ↓ CLs	Irregular		(40)
IR/LepR^POMC^ (mouse)	↑	IR, ↑ insulin, glucose intolerance	↑ T	↑ LH	Infertile	Occasional cyst-like follicles	Acyclic, anovulatory		(41)
Neuron-specific *IRS2* deletion (mouse)	↑	↑ FBG, glucose intolerance	↓ T	↓ LH	Infertile	Small ovaries, ↓ total follicle numbers	Acyclic, anovulatory		(42)
			↓ E2						
*AKT2* deletion (mouse)	→	↑ insulin (older mice only)	↑ T (older mice only)	LH normal	Young mice fertile	Large luteinized cysts	N/R	Mice aged 120 weeks.	(43)

*CL, corpus lutea; E2, estradiol; FBG, fasting blood glucose; FSH, follicle-stimulating hormone; IR, insulin resistance; LH, luteinizing hormone; N/R, not reported; P, progesterone; PCOS, polycystic ovary syndrome; T, testosterone; T2DM, type 2 diabetes mellitus; TGs, triglycerides*.

## Key Players in PCOS Pathogenesis

### Metabolic Features of PCOS

While PCOS is robustly associated with impaired insulin sensitivity and hyperinsulinemia (Table 1), this is independent of body weight, and a significant proportion of insulin-resistant women with PCOS are lean (44, 45). However, it is recognized that increased body weight exacerbates hyperandrogenism, oligomenorrhoea, and metabolic risk in PCOS (46, 47), and genetic studies have revealed a role for obesity-associated genes (48, 49).

Several observations suggest that IR, and associated compensatory hyperinsulinemia, may play a key pathogenic role in PCOS. Firstly, IR is more common in women with both hyperandrogenism and anovulation, compared to weight-matched hyperandrogenemic women with normal ovulatory cycles (50). Second, interventions that increase insulin sensitivity improve, independent of weight loss, ovulatory function, menstrual cyclicity, fertility, and hyperandrogenism in lean and obese patients (51–55). Third, a severe PCOS-like syndrome is a prominent (often-presenting) feature in patients with severe, genetic forms of IR (56) and is also reportedly associated with pancreatic insulinomata and excessive exogenous insulin in type 1 diabetes (57, 58).

Importantly, PCOS likely represents a state of “partial” IR, in which preserved insulin signaling in ovarian theca cells causes excessive androgen synthesis and theca cell proliferation, with subsequent hyperandrogenemia (Figure 1) (59–62). Other potential effects of hyperinsulinemia include reduced hepatic synthesis of sex hormone-binding globulin, thereby increasing free testosterone, hypersecretion of pituitary luteinizing hormone (LH), and reduced insulin-like growth factor-binding protein (63–65). This latter effect potentially modulates the paracrine growth factor-dependent regulation of early follicle development and dominant follicle selection (Figure 1).

### Ovarian Dysmorphology

The abnormal appearance of the ovarian cortex in PCOS represents inappropriate and excessive initiation of follicle growth from the primordial follicle pool, followed by developmental failure and growth arrest at the medium-sized antral stage (5–10 mm) (66–68). Loss of coordinated follicle development results in fewer or absent ovulations, and therefore subfertility. Histologically, the ovary contains a reduced number of corpora lutea (representing fewer ovulations), more atretic follicles, stromal hypertrophy, and increased ovarian weight. As mentioned, hyperthecosis is prominent, with *in vitro* evidence suggesting that abnormal thecal cell proliferation contributes to excessive androgen biosynthesis (62, 69).

### Hormonal Dysregulation

While IR and hyperinsulinemia may play a central, and in some cases primary, role in PCOS pathogenesis, the importance of hyperandrogenism should be stressed. Not only is it a defining feature of the syndrome, both in ovulatory and anovulatory women, but other conditions associated with excessive androgen exposure (such as congenital adrenal hyperplasia and androgen-secreting tumors) also produce features of PCOS (70). Moreover, administration of androgens in rodents, sheep, and non-human primates results in pathophysiological changes that closely resemble features of PCOS in women. Androgens act at the ovary to disrupt follicular development and dominant follicle selection by promoting excessive early follicular growth, while systemic effects include development of IR and metabolic dysfunction (71–77). The role of androgens in PCOS may be particularly important during key developmental windows before the onset of IR (13). Prenatally, androgenized rhesus monkeys and sheep demonstrate ovarian hyperandrogenism and IR in adulthood, with increased follicle numbers, anovulation, and LH hypersecretion (78–81).

Dysregulation and reprograming of the hypothalamus–pituitary–ovarian (HPO) axis is common in PCOS, potentially driven by androgen exposure *in utero* and manifesting as hypersecretion of LH, persistently rapid LH pulse frequency, and below-normal levels of follicle-stimulating hormone (FSH) (82, 83). These alterations likely contribute to disrupted follicle development in PCOS, while high levels of LH also synergize with insulin to promote theca androgen production (Figure 1). However, it is noteworthy that many patients have normal LH levels, suggesting that elevated gonadotrophin levels is unlikely to be the primary defect in PCOS (84).

## Ovarian Dysfunction in Genetic Models of Metabolic Disease

### Rodent Models of Obesity

While there is no spontaneously occurring animal model of PCOS, transgenic and knockout rodent models widely used in metabolic research provide opportunities to study specifically the association between metabolic disease and ovarian dysfunction. However, it is important to note that key differences exist between human and rodent ovarian function. Whereas in humans, full follicular differentiation occurs in the later stages of fetal development, in rodents this occurs postnatally. The mouse estrus cycle lasts only 4–6 days, compared to 28 days in humans. Furthermore, rodents are polyovulatory, suggesting important differences in dominant follicle selection despite underlying similarities in the HPO axis.

In spite of these differences, various rodent models of obesity do display reproductive phenotypes comparable to PCOS (Table 1). Diet-induced obesity in wild-type mice is associated with disrupted estrus cyclicity, fewer corpora lutea, reduced fertility, and metabolic dysfunction, supporting the notion that obesity-associated metabolic dysfunction may contribute to PCOS (24, 25, 85). Among the genetic models, female *ob/ob* and *db/db* mice, which, due to loss-of-function mutations in leptin and leptin receptor, respectively, are hyperphagic, severely obese, hyperinsulinemic, and hyperglycemic are also infertile, acyclic, and anovulatory (Table 1). Morphologically, they show utero-ovarian atrophy, follicular atresia, apoptotic granulosa cells, deformed oocytes, absent corpora lutea, and no cystic structures (26, 27, 29–32). The endocrine profile of *ob/ob* mice includes elevated serum testosterone, estradiol, and progesterone, with reduced FSH but normal LH, while *db/db* mice have low estradiol and progesterone. The obese Koletsky and Zucker diabetic fat rats, both of which also lack functional leptin receptors, do exhibit estrus cycling (albeit irregularly) but are subfertile with increased follicle numbers, follicular atresia, and fewer corpora lutea. While androgen levels are elevated in the obese Koletsky, in Zucker, they are reportedly below normal (33–36). The New Zealand obese (NZO) mouse, notable for being a polygenic model of the human metabolic syndrome (37), also harbors leptin receptor variants and displays a reproductive phenotype similar to that of Zucker (Table 1) (38, 39).

In all of these models, reproductive dysfunction is at least partly attributable to loss of hypothalamic leptin signaling, rather than obesity *per se*. Genetic leptin deficiency in humans is associated with low gonadotrophins and pubertal failure, which are restored with leptin replacement (86). Fertility, litter size, and estrus cyclicity of *ob/ob* mice were similarly ameliorated by human recombinant leptin (87, 88) or transplantation with wild-type adipose tissue (89, 90). Along with other peripheral signals, leptin is believed to modulate the activity of gonadotrophin-releasing hormone (GnRH) releasing neurons – and thus the entire HPO axis – in response to nutritional status (91). Indeed, low body weight is known to interfere with reproductive function and pubertal timing (92). Failure of central leptin action in rodent models of obesity therefore leads to infertility due to hypogonadotrophic hypogonadism and follicle development (Figure 1). Indeed, human obesity is also associated with a degree of hypothalamic leptin resistance, which may contribute to HPO dysregulation in PCOS (93, 94). Reports of excess lipid accumulation in follicular cells of *ob/ob* mice and the obese Koletsky rat suggest an additional “lipotoxic” mechanism by which extreme obesity may produce ovarian dysfunction, although there are no reports of such a phenotype in PCOS (30, 36).

In these models, the relative contribution of IR-associated hyperinsulinemia and central leptin resistance is difficult to disentangle, particularly since hypothalamic insulin signaling also regulates GnRH release and thus reproduction function (40, 41, 95–97). Mice with neuron-specific deletion of the insulin receptor gene (*Insr*) or hypothalamic POMC neuron-specific deletion of both leptin and *Insr* were hyperphagic, insulin resistant, and subfertile due to impaired follicular development (Table 1) (40, 41, 98). The combined knockout was notable for high levels of LH, hyperandrogenemia, and cyst-like follicles. POMC-specific deletion of leptin receptor alone produced only a subtle reproductive phenotype (99). Counterintuitively, pituitary-specific *Insr* knockout reportedly *rescued* the PCOS-like phenotype associated with diet-induced obesity (24). These observations highlight complex interactions between leptin and insulin in their regulation of reproductive function. Indeed, studies in mammals and non-mammalian species reveal that nutritional status and reproductive capacity are tightly intertwined, ensuring that reproduction only proceeds if nutritional status is optimal (100).

### Genetic Models of Insulin Resistance

In humans, rare loss-of-function mutations in *INSR* not only cause extreme hyperinsulinemia but also oligomenorrhoea, hyperandrogenism, and excessive development of sex hormone-dependent tissues (56). Common genetic defects in insulin signaling are suggested to contribute to PCOS heritability (101, 102), and cellular studies reveal abnormalities in insulin-mediated insulin receptor autophosphorylation, IRS expression, PI3-kinase activation, GLUT4 expression, and insulin-stimulated glucose uptake in adipocytes and skeletal muscle from women with PCOS (103–107). However, the results of such studies are variable and need further verification.

Mice lacking functional insulin receptor develop profound metabolic abnormalities at birth and die within days. Of the tissue-specific knockouts, only those targeting the brain have a reported reproductive phenotype (108). Similar to the neuron-specific *Insr* knockout, global deletion of *Irs2* (but not *Irs1*) causes a combination of metabolic, reproductive, and ovarian features that likely result from disrupted central insulin and leptin action rather than abnormal systemic glucose metabolism (42) (Table 1). Thus, in addition to the impact of systemic hyperinsulinemia, the interpretation of global insulin signaling defects must consider the actions of insulin at the hypothalamus as well as disruption to the regulation of early follicle development by IGF1. There are no corresponding human syndromes of IRS dysfunction or deficiency with which to compare.

Downstream of IRS in the signaling pathway, non-functional mutations in human *AKT2* result in ovarian hyperandrogenism in the context of partial lipodystrophy, severe IR, diabetes, metabolic dyslipidemia, and fatty liver (109). In mice, global *AKT2* deletion produced a somewhat comparable ovarian phenotype, with increased androgenic steroidogenesis in the theca-interstitium, theca-interstitial hyperplasia, hyperandrogenemia, reduced corpora lutea, and ovarian cysts but normal LH levels (Table 1) (43). However, the large, luteinized, serous-filled cysts were quite distinct from the ovarian morphology characterizing human PCOS. For unclear reasons, reproductive features were absent in younger mice, although could be induced by treatment with LH, perhaps due to synergism with hyperinsulinemia.

Other human lipodystrophy syndromes (genetic or acquired) are similarly characterized by severe IR, ovarian hyperandrogenism, amenorrhea, and infertility (110–112). While genetic mouse models of generalized lipodystrophy manifest many metabolic features of the human diseases, “partial” lipodystrophy has been more challenging to model (113). Moreover, while the metabolic properties of these models have been interrogated in detail, their reproductive and ovarian phenotypes have not been reported widely. Studying these models may provide important new insights into the role of BMI-independent IR in PCOS-like ovarian dysfunction.

### Genetic Models Targeting the HPO Axis

To better understand PCOS pathogenesis, rodent models of obesity and IR should be considered alongside those in which other implicated systems are targeted. Transgenic mice with chronically elevated gonadotrophin levels have a thickened theca cell layer, similar to PCOS, with correspondingly increased estrogen and testosterone levels (23, 114). However, unlike PCOS, their ovaries contain large, hemorrhagic cysts, as do those of mice lacking LH receptor (114, 115). Global or theca-specific deficiency of estrogen receptor subunits ERα or ERβ, or global deficiency of aromatase, produces chronically elevated gonadotrophins (due to lack of estradiol), arrested follicular growth, absent corpora lutea and anovulation (116–118). ERα knockout mice also show increased adiposity (without hyperphagia), IR, and diabetes (118, 119), whereas constitutive elevation of LH activity produces hyperphagic obesity with hyperleptinemia and hyperinsulinemia (120). These observations further illuminate the complexity of nutritional and reproductive cross talk in humans, again challenging the value of simplified rodent models.

## Transgenic Rodents and PCOS – Not Fit for Purpose?

This discussion reveals that transgenic models of PCOS are complex, heterogeneous, and even the best examples deviate in important ways from the human syndrome. Models of obesity and IR have not typically been studied comprehensively from a reproductive perspective. Even when a reproductive deficit is noted, the ovarian and endocrinological phenotyping is often incomplete, with concerns raised over timing of the studies (relative to time of day, phase of the estrous cycle, and age of the animal), the rigor of morphological analyses, and the variability of ovarian appearances described as “cystic.” Furthermore, as outlined above, important differences exist between human and rodent ovarian function. Such differences may explain why the reproductive consequences of androgen exposure are less consistent in rodents than in sheep or primates, and emphasize that results from rodent-based studies (genetic or hormonal) need to be extrapolated with caution to human PCOS (13, 23).

Emerging from this discussion is an important reminder that reproductive capacity and nutritional status are intertwined tightly through feedback and cross talk between reproductive and metabolic pathways. Across a wide range of species, including *Caenorhabditis elegans* and *Drosophila*, conserved mechanisms operate to regulate reproduction and energy homeostasis (121–123). In rodent models of obesity, the same lesions that produce hyperphagia also directly impact on the HPO axis, thereby complicating their interpretation. The bidirectional interaction between reproductive and nutritional signaling also operates systemically: while estrogen drives adipogenesis, and while testosterone drives food intake, both steroids in excess produce IR, hyperinsulinemia, high levels of circulating leptin, and reduced levels of adiponectin, all of which impact on the HPO axis and ovarian function. The hope of mimicking this complex network by perturbing single or a few genes is perhaps ambitious. Indeed, the notion that PCOS is precipitated by a single etiological factor is undoubtedly too simple. While monogenic perturbations in insulin signaling or adipose function in humans do produce PCOS-like syndromes, differences between human and rodent metabolism and reproduction mean that PCOS will not necessarily emerge from equivalent defects in mice. As in all complex human disease, the role of genetic, developmental, and environmental factors likely contribute heavily to the heterogeneity of human PCOS.

## Future Opportunities

Complementary strategies are required to better understand this growing health problem. The combined use of hormonal treatments in transgenic animals may afford interesting, clinically relevant insights. Primary follicular cell and whole follicle cultures, including from transgenic animals, facilitate the study of tightly regulated paracrine and autocrine networks in early follicle development that become disordered in PCOS (124). The ease and efficiency of CRISPR-Cas9-based gene editing technologies will doubtless prove invaluable, particularly to explore new susceptibility loci emerging from large GWAS studies (48, 49, 101, 102, 125). Many of these loci implicate genes of largely unknown function. As they are investigated over the coming years, prudent selection of appropriate cell and animal systems will be imperative. The study of candidate genes in non-ovarian cell types is questionable, yet primary cultures are difficult to acquire and maintain, and ovarian cell lines are too atypical in their properties to be useful. Therefore, in spite of reservations highlighted above, transgenic rodent models will likely play an ongoing role in our effort to better understand and manage this challenging condition.

## Conclusion

A clear relationship exists between obesity, metabolic dysregulation, and ovarian dysfunction. However, the mechanisms of this association are poorly understood. Without detailed knowledge of the etiology of PCOS, management is limited to empirical and symptomatic treatment. While hormonal models of PCOS demonstrate an important role for hyperandrogenemia, the reported genetic models incompletely replicate the PCOS phenotype. Their study has offered important insights into the interaction between metabolism and reproduction, but clear conclusions about PCOS pathogenesis have not been forthcoming. Nevertheless, specific models may prove useful for answering reductionist questions about aspects of the condition, such as disordered folliculogenesis or disruption of the HPO axis. Future efforts will benefit from ongoing combined study of humans, mouse models, and cells, driven by insights emerging from human genetic studies. These studies will continue to advance our understanding of this important condition and, with time, support new approaches to addressing both the metabolic and reproductive problems faced by affected women.

## Author Contributions

Both IH-D and SF contributed to the content, writing, and editing of this manuscript.

## Conflict of Interest Statement

The authors declare that this article was written in the absence of any commercial or financial relationships that could be construed as a potential conflict of interest.

## Abbreviations

FSH, follicle-stimulating hormone; GnRH, gonadotrophin-releasing hormone; IR, insulin resistance; LH, luteinizing hormone; PCOS, polycystic ovary syndrome; T2DM, type 2 diabetes mellitus.
